# Construction of Yeast One-Hybrid Library of *Dendrobium huoshanense* and Screening of Potential Transcription Factors Regulating *DhPMM* Gene Expression

**DOI:** 10.3390/biom15091251

**Published:** 2025-08-29

**Authors:** Jing Wu, Shuting Wang, Shihai Xing, Daiyin Peng

**Affiliations:** 1 College of Pharmacy, Anhui University of Chinese Medicine, Hefei 230012, China; 2020205225051@stu.ahtcm.edu.cn (J.W.); 2020205225011@stu.ahtcm.edu.cn (S.W.); 2 MOE-Anhui Joint Collaborative Innovation Center for Quality Improvement of Anhui Genuine Chinese Medicinal Materials, Hefei 230012, China; 3 Institute of Traditional Chinese Medicine Resources Protection and Development, Anhui Academy of Chinese Medicine, Hefei 230012, China

**Keywords:** *Dendrobium huoshanense*, phosphomannomutase, yeast one-hybrid assay, transcription factors, polysaccharide biosynthesis

## Abstract

*Dendrobium huoshanense*, an endangered orchid species, is renowned for its polysaccharides with vast pharmacological significance in stems. Phosphomannomutase (PMM) critically regulates polysaccharide accumulation. Transcriptional regulation of *DhPMM* remains poorly characterized. This study employed a yeast one-hybrid (Y1H) system to identify upstream regulators of *DhPMM*. The 2.15 kb *DhPMM* promoter was cloned, revealing multiple stress- and hormone-responsive cis-elements (e.g., ABRE, MYC, ERF). A high-complexity Y1H library (3.60 × 10^9^ CFU) was constructed with insert sizes averaging 1–2 kb. Screening using aureobasidin A (AbA)-resistant Y1HGold [pAbAi-*DhPMM*] identified 11 candidate clones, including four transcription factor families (DOF, NAC, ERF, BES1). Interactions were rigorously confirmed by pairwise Y1H showing AbA-resistant growth and dual-luciferase assays demonstrating *DhPMM* activation. This represents the first functional cDNA library resource for *D. huoshanense* and identification of TFs interacting with *DhPMM*. The discovery of TFs belonging to DOF, NAC, ERF, and BES1 families as *DhPMM* regulators elucidated the transcriptional network underlying polysaccharide biosynthesis. This establishes a transcriptional framework for engineering polysaccharide biosynthesis in *D. huoshanense*.

## 1. Introduction

*Dendrobium huoshanense* C. Z. Tang et S. J. Cheng, classified within the second largest genus in the family Orchidaceae, is a precious traditional Chinese materia medica [1]. Due to its outstanding medicinal values, it was officially compiled in the Chinese Pharmacopeia as expected in 2020 [2]. The stems of *D. huoshanense* traditionally used as the medicinal part have shown significant benefits in disease prevention and treatment [3]. Studies have revealed that there are numerous active components in the stem, among which polysaccharide has been the most extensively studied [4,5]. However, wild *D. huoshanense* populations face critical endangerment due to habitat loss and excessive harvesting despite high market demand accompanied by its listing as a national first-class protected species [6]. Understanding the molecular mechanisms governing polysaccharide biosynthesis is therefore essential for both enhancing metabolite production through metabolic engineering and developing sustainable cultivated germplasm.

Enzymes and their corresponding encoding genes have irreplaceable roles in polysaccharide biosynthesis and metabolism pathways [7]. Phosphomannomutase (PMM), which catalyzes the isomerization of mannose-6-phosphate to mannose-1-phosphate, represents a rate-limiting enzyme in GDP-mannose synthesis—the universal precursor for plant polysaccharides [8]. He et al. [9] conducted transcriptional analyses of *DoPMM* gene from *Dendrobium officinale*. *DoPMM* showed peak expression levels in stems. Similarly, *CpPMM* expression in *Codonopsis pilosula* showed spatiotemporal differentiation positively associated with polysaccharide content [10]. *D. huoshanense* exhibited analogous tissue-specific *DhPMM* expression patterns, with the highest transcript levels occurring in stems [11]. Collectively, these studies confirm PMM as a phylogenetically conserved regulator of polysaccharide biosynthesis in medicinal plants [8].

Transcriptional regulation of metabolite biosynthesis occurs through *cis*-elements within enzyme gene promoters. These regulatory sequences serve as binding sites for transcription factors (TFs) that modulate gene expression in response to developmental and environmental stimuli [12]. TFs play pivotal roles in regulating plant metabolite biosynthesis through enzyme-mediated pathways in response to external signals [13]. Accumulating evidence demonstrates that overexpression of TFs is an effective strategy to increase sugar and starch contents [14,15]. The yeast one-hybrid (Y1H) system has been developed as a tool to screen prey proteins interacted with bait DNA promoter sequences, enabling TF identification [16]. Successful applications of Y1H screening have identified candidate TFs regulating secondary metabolites biosynthesis in medicinal species including *Madagascar periwinkle* [17], *Taxus chinensis* [18], *Ganoderma lucidum* [19], and *Artemisia annua* [20]. However, systematic investigations on transcriptional networks governing pharmacologically valuable polysaccharide biosynthesis in *D. huoshanense* remain unreported.

Here, we isolated the *DhPMM* promoter, constructed a Y1H bait vector, and identified TFs binding this regulatory sequence. Our findings may not only contribute to further understanding of the role of PMM in polysaccharide biosynthesis, but also provide validated molecular targets for metabolic engineering to enhance polysaccharide production in *D. huoshanense.*

## 2. Materials and Methods

### 2.1. Plant Materials

*Dendrobium huoshanense* C. Z. Tang et S. J. Cheng, cultivated under forest from Huoshan County, Anhui Province, China was identified by Professor Nianjun Yu from Anhui University of Chinese Medicine [21]. The other type of greenhouse-cultivated *D. huoshanense* was also collected and mixed up with that under forest for the samples. Voucher specimens of plants were deposited at the Herbarium Center, Anhui University of Chinese Medicine, Hefei, China (Daiyin Peng, Pengdy@ahtcm.edu.cn, Voucher Nos. 20221104 and 20221105). *Nicotiana benthamiana* was grown in chambers (22 °C, 16 h light/8 h dark cycle) with three-week-old plants used for transient expression assays. No permission or license was required in this study. The sample was legally collected in accordance with guidelines provided by the national or international regulations. Field studies complied with local legislation.

### 2.2. Cloning of the Promoter of Gene DhPMM and Cis-Element Analysis

Genomic DNA was isolated from leaves of samples with Ezup Column Plant Genomic DNA Kit (B518262–0050, Sangon Biotech, Shanghai, China). The *D. huoshanense* genome was searched in the NCBI database (https://www.ncbi.nlm.nih.gov/, accessed on 15 July 2025) [22] for the ATG upstream sequence of *PMM* gene promoter [23]. Primers were designed to amplify this sequence by polymerase chain reaction (PCR) amplification, and products were verified by 1% agarose gel electrophoresis and sequencing. Predicted cis-acting elements within the promoter were analyzed using PlantCARE (http://bioinformatics.psb.ugent.be/webtools/plantcare/html/, accessed on 15 May 2024) [24].

### 2.3. Construction of a Recombinant pAbAi-DhPMM Bait Plasmid

The vector pAbAi provided by OEbiotech (Shanghai, China) [25] and purified *DhPMM* promoter fragment were digested concurrently with *SacI* and *XhoI*. Digested fragments were gel-purified using PureLink Quick Gel Extraction Kit (K220001, Invitrogen, Carlsbad, CA, USA) and ligated using T4 DNA Ligase (EL0014, Thermo Fisher Scientific, Waltham, MA, USA). The ligation product was transformed into *Escherichia coli* (*E. coli*) DH10β competent cells and plated on Luria–Bertani (LB) agar (ampicillin resistant) to screen positive recombinant clones.

### 2.4. Generation of the Yeast Bait Strain

The recombinant pAbAi-*DhPMM* plasmid was linearized with *BstBI*. Y1HGold yeast competent cells were transformed with 1 μg linearized DNA via the lithium acetate/single-stranded carrier DNA/polyethylene glycol (LiAc/SS-DNA/PEG) method [26]. Transformants were selected on solid agar synthetic defined (SD)/-Ura plates at 30 °C for 72 h. Individual colonies were screened by PCR to confirm genomic integration of the bait construct. Aureobasidin A (AbA) sensitivity was assayed by spotting serial dilutions of a saturated culture of a single PCR-confirmed bait strain onto SD/-Ura plates containing AbA at concentrations of 0, 100, 150, 200, 300, 500, 700, and 900 ng/mL. The minimal AbA concentration that completely inhibited growth after 3 days at 30 °C was selected for library screening.

### 2.5. Construction of the Prey cDNA Library

Total RNA was isolated from pooled greenhouse- and forest-cultivated *D. huoshanense* tissues using RNAiso Plus (9108Q, Takara, Kyoto, Japan) combined with Fruitmate for RNA purification kits (9192, Takara). mRNA was enriched according to FastTrack MAG mRNA Isolation Kit (K1580-02; Invitrogen). After that, first-strand cDNA synthesis was performed using the SMART™ technology (CloneMiner™ cDNA Library Construction Kit, A11180, Invitrogen), incorporating attB adapters via gene-specific primers during PCR amplification of ds cDNA. Purified ds cDNA was size fractionated using a CHROMA SPIN-1000-TE (Clontech, Mountain View, CA, USA). The attB-flanked ds cDNA library was recombined into the pDONR222 vector via BP Clonase II Enzyme Mix (Thermo Fisher Scientific) to generate the primary library plasmid. Then the secondary cDNA library was constructed by LR recombination reaction. The primary library plasmids were cloned into pGADT7-DEST and incubated with LR Clonase II enzyme mix (Invitrogen) [27] to create the AD fusion prey expression library (pGADT7-Rec plasmid). The final normalized prey plasmid library was amplified in *E. coli* competent cells DH10β and plasmid DNA was extracted using the PureLink HQ Kit (K210001, Invitrogen) for subsequent experiments.

### 2.6. Screening of a Y1H Library

The 5 μg normalized pGADT7 cDNA prey library was transformed into the AbA-sensitive bait strain (Y1HGold [pAbAi-*DhPMM*]) via the LiAc/SS-DNA/PEG method. Transformants were resuspended in 0.9% NaCl and subjected to serial 10-fold dilutions (10^0^–10^−3^) and 100 μL diluents were coated onto SD-Leu plates to calculate the titer and the number of colonies: total number of colonies = colony-forming units (CFU)/(volume plated × dilution factor) × total suspension volume (mL) [28]. Then, yeast cell suspensions were coated on corresponding SD-Leu/AbA (containing the predetermined minimal inhibitory concentration of AbA).

### 2.7. Identification and Sequencing of Positive Clones

Putative positive clones from the primary screening were restreaked onto SD/-Leu/AbA plates (300 ng/mL AbA) for 72 h as the secondary screening to eliminate false positives. Surviving clones were cultured in SD/-Leu liquid medium at OD_600_ = 0.6–0.8 and validated by the Matchmaker Insert Check PCR Mix 2 (630497, Takara). Purified plasmids were extracted and Sanger-sequenced with vector-specific primers.

### 2.8. Pairwise Validation of the Promoter of DhPMM and Candidate TFs

After all the positive clones were analyzed by BLASTn (v2.17.0) against NCBI, several typical TFs were screened out. To validate interactions with the *DhPMM* promoter, pairwise yeast one-hybrid retransformation assays were performed. Competent Y1HGold [pAbAi-*DhPMM*] bait cells were generated using the LiAc/SS Carrier DNA/PEG method. Prey plasmids encoding candidate TFs were extracted from yeast clones. Purified prey plasmids (200 ng) were individually transformed into Y1HGold [pAbAi-*DhPMM*] strains. Transformants were plated on SD/-Leu and SD/-Leu/AbA (300 ng/mL AbA). TF-*DhPMM* promoter interactions were confirmed by growth on the SD/-Leu/AbA plates.

### 2.9. Dual-Luciferase (Dual-LUC) Assay

Putative TF-promoter interactions identified by Y1H screening were independently verified through dual-LUC assays to exclude false positives [29]. Target promoter sequences of *DhPMM* were cloned into the pGreenII 0800-LUC reporter vector, while candidate TFs were inserted into the pGreenII 62-SK effector vector, which were separately transformed into *Agrobacterium tumefaciens* GV3101. Vectors were part of our laboratory’s established vector collection. Bacterial cultures were centrifuged, resuspended in MES buffer to an OD_600_ = 0.6, and dark-adapted for 3 h at 22 °C. Effector strains (pGreenII 62-SK-TFs) and reporter strains (pGreenII 0800-*DhPMM-pro*-LUC) were combined at a 1:1 ratio, with pGreenII 62-SK empty vector controls. Suspensions were infiltrated into *N. benthamiana* leaves using 1 mL needless syringes. After 48 h of incubation, LUC imaging was detected using a chemiluminescence imaging system (IVIS Lumina Series, PerkinElmer, Waltham, MA, USA). LUC/Renilla (Ren) activity ratios were quantified using the Dual Luciferase Reporter Gene Assay Kit (KTA8010, Abbkine, Mountain View, CA, USA) with three biological replicates performed. Statistical analyses were performed with Bonferroni correction for multiple testing correction [30]. For dual-luciferase assays testing 4 transcription factors, the significance threshold was adjusted to 0.0125.

## 3. Results

### 3.1. Cloning of DhPMM Gene Promoter

**Figure 1:**
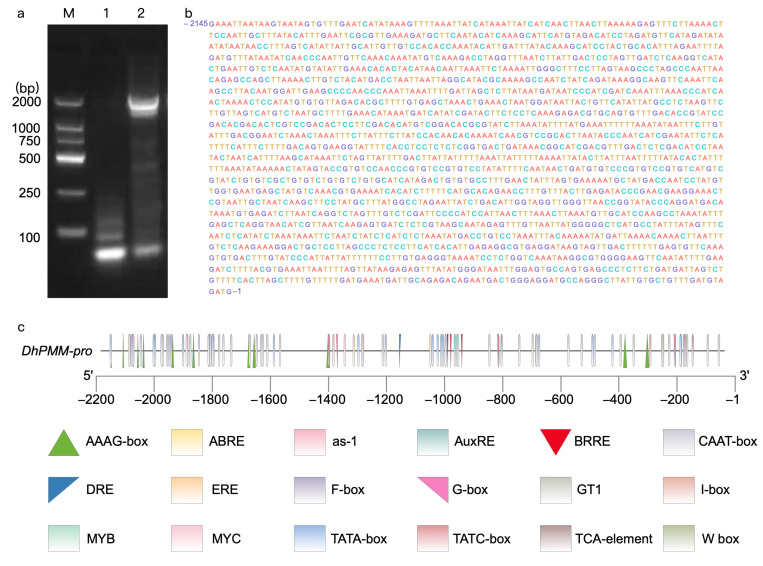
Cloning of the promoter of *DhPMM.* (**a**) M: DNA marker, lane 1: the blank control, lane 2: the amplified fragment of the promoter of *DhPMM*. (**b**) Sequencing results of cloned promoter of *DhPMM.* (**c**) *Cis*-elements in the sequence of the promoters.

Using *D. huoshanense* genomic DNA as template, PCR amplification and agarose gel electrophoresis were performed to isolate the *DhPMM* promoter sequence (Figure 1a). The PCR product was cloned into pMD19-T vector and transformed into *E. coli* DH5α, among which positive clones were verified and sequenced with a length of 2145 bp (Figure 1b). The promoter sequences were analyzed on the online website PlantCARE, which showed that varieties of *cis*-elements were involved such as ABRE, CAAT-box, MYC, and other uncharacterized regions (Table 1, Figure 1c).

### 3.2. Identification of Bait Yeast Strain and Determination of AbA Basal Expression

**Figure 2:**
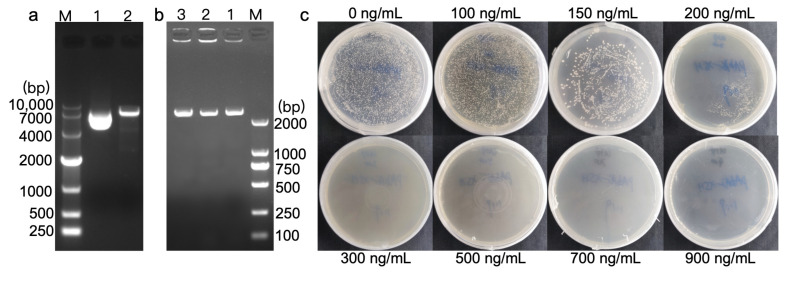
Bait strain validation and AbA autoactivation determination. (**a**) *BstBI* digestion of pAbAi-*DhPMM*. Lane 1: undigested plasmid, lane 2: digestion products (4.87 kb vector + 2.15 kb insert). (**b**) PCR verification of genomic integration of linearized pAbAi-*DhPMM* in Y1HGold (397 bp + 2145 bp). Lanes 1–3: three positive clones. (**c**) Determination of the minimum inhibitory AbA concentration for Y1HGold [pAbAi-*DhPMM*] on the SD/-Ura plates.

The *DhPMM* promoter was cloned into pAbAi via restriction digestion, generating recombinant pAbAi-*DhPMM*. *BstBI* digestion yielded fragments of 7000 bp, consistent with the expected size (2145 bp insert + 4870 bp vector, Figure 2a). Finally, PCR identification confirmed the bait plasmid pAbAi-*DhPMM* was successfully transformed into Y1HGold competent cells (Figure 2b).

Prior to cDNA library screening on SD/-Leu/AbA plates, the bait strain’s autoactivation potential was assessed to ascertain that interacting proteins were derived from the library rather than endogenous yeast proteins [31]. Figure 2c illustrated that strains grew vigorously on plates with AbA concentrations of 0, 100, 150, and 200 ng/mL, but showed complete growth inhibition at 300 ng/mL. Thus, the minimum AbA concentration 300 ng/mL was established for subsequent cDNA library screening.

### 3.3. cDNA Library Construction and Validation

The integrity of mRNA and ds cDNA was confirmed by agarose gel electrophoresis, on which bands showed continuous smears within 0.5–5 kb, (Supplementary Figure S1a,b). The primary library (pDONR222-cDNA) exhibited a volume of 1.44 × 10^7^ CFU with 100% recombination efficiency and ≥1 kb inserts, as determined by colony PCR analysis of 24 randomly selected clones (Supplementary Figure S2a,b). The second library (pGADT7-cDNA) showed a volume of 1.20 × 10^7^ CFU and consistent insert lengths (Supplementary Figure S2c,d). The results of volumes and recombination rates of the cDNA library stood for the favorable capacity and quality of the constructed library.

### 3.4. Screening of the Yeast One-Hybrid Library and Plasmids Extraction

**Figure 3:**
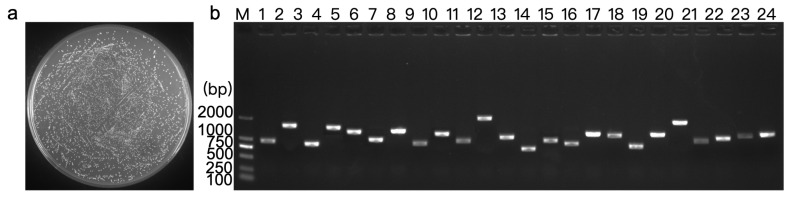
Quality assessment of the construction of the pAbAi-*DhPMM* Y1H library. (**a**) pAbAi-*DhPMM* Y1H library met stringent quality criteria (CFU = 3.60 × 10^9^). (**b**) Twenty-four clones were randomly chosen to determine the average size of the inserted fragments using colony PCR. Lanes 1–24: 24 randomly selected clones.

The constructed Y1H expression library (pGADT7-cDNA) plasmid of *D. huoshanense* was introduced into the Y1HGold [pAbAi-*DhPMM*]. There were 1200 clones growing on the SD/-Leu plate with a titer of 1.20 × 10^7^ CFU/mL (Figure 3a). The total number of screened clones was calculated as 3.60 × 10^9^. Colony PCR and electrophoretic identification declared insert fragments ranging from 1 kb to 2 kb in length (Figure 3b).

**Figure 4:**
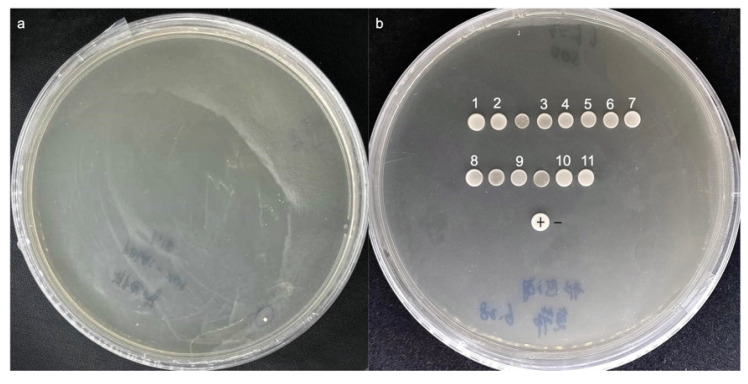
Screening of the pAbAi-*DhPMM* Y1H library. Growth of positive mono-clone in SD/-Leu/AbA (300 ng/mL) solid medium (**a**) and their secondary culture (**b**).

Y1H system represents a well-established strategy for identifying transcriptional regulatory roles. However, it is associated with a high frequency of false positives and sequence replication issues, which can be mitigated by employing two rounds of screening [32]. Following the first screening, 14 clones tested positive were found on the SD/-Leu/AbA (300 ng/mL) plate (Figure 4a). Eleven of the fourteen positive clones underwent further growth after being transferred on the SD/-Leu/AbA (300 ng/mL) medium for a second screening (Table 2, Figure 4b). Ultimately, following sequencing and identification, the roles and functions of the 11 identified genes were clarified. Among these sequences, four were annotated as transcription factors based on domains identification: no apical meristem, *Arabidopsis thaliana* transcription activation factor, cup-shaped cotyledon (NAC), DNA binding with one C2-C2 zinc finger domain (DOF), ethylene-responsive factors (ERF), and Bri1-ems-suppressors (BES1) families (Table 2).

### 3.5. Validation of the Interaction of the Promoter of DhPMM and Candidate TFs

**Figure 5:**
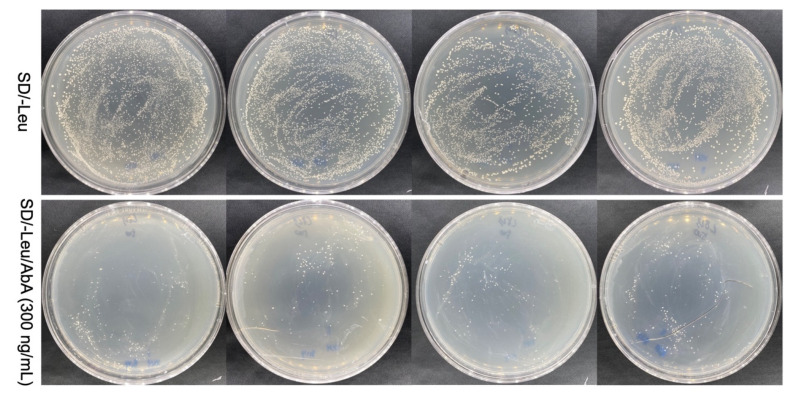
Validation of candidate transcription factors interacting with the promoter of *DhPMM* in the SD/-Leu/AbA (300 ng/mL) solid medium. From left to right are positive colonies identified as NAC, DOF, ERF, and BES1 families.

*DhPMM* serves as a key catalytic enzyme in the polysaccharide biosynthesis pathway of *D. huoshanense* [33], while no transcription factor has been reported to regulate the expression of the *DhPMM* gene. Therefore, we focused on four TFs among those 11 candidate sequences to further confirm whether there were interactions between the candidate TFs with the 5′ transcriptionally active region (−2145 to −1 bp) of *DhPMM*. Each TF was individually transformed into Y1HGold [pAbAi-*DhPMM*] for one-to-one verification. All transformants were able to grow and spread on SD/-Leu plates, as demonstrated by the findings (Figure 5), suggesting that the Y1H transformation system was working well. Crucially, strains of D1, D2, D4, and D6 belonging to TFs families demonstrated robust growth on SD/-Leu/AbA (300 ng/mL) plates. The AbA-resistant growth of these four TFs on the plates indicated specific protein–DNA interactions.

### 3.6. NAC, DOF, ERF, and BES1 Interact with the Promoter of the DhPMM and Activate Its Expression

**Figure 6:**
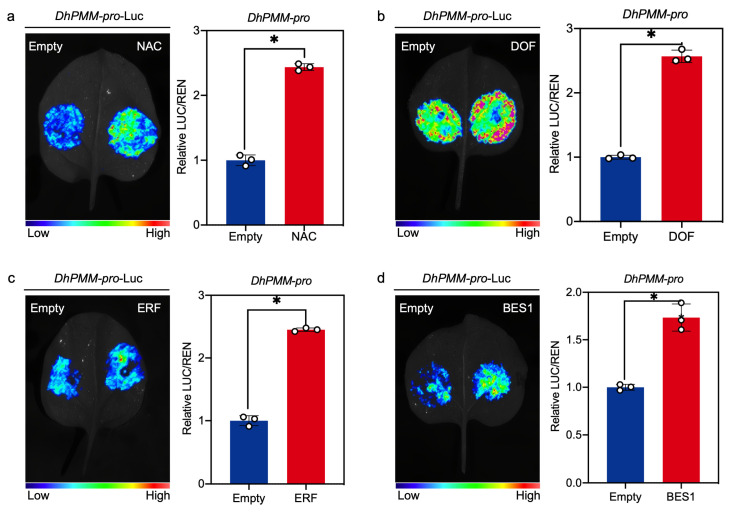
Interaction of TFs belonging to NAC, DOF, ERF, and BES1 families with *DhPMM* promoter detected by dual-LUC assay. Luminescence imaging analysis of tobacco leaves and the determination of LUC/REN relative activity ratio of the pGreen-*DhPMM-pro*-LUC and pGreenII 62-SK-TFs ((**a**) NAC, (**b**) DOF, (**c**) ERF, and (**d**) BES1). Empty vector pGreenII 62-SK and reporter pGreen-*DhPMM-pro*-LUC functioned as negative control. Data represent mean ± SD (n = 3). Bonferroni correction: * *p* < 0.0125.

In addition, dual-luciferase assays were performed to verify the interactions between the *DhPMM* promoter and four candidate TFs from NAC, DOF, ERF, and BES1 families identified via Y1H screening. Co-expression of TF effectors (pGreenII 62-SK-TFs) with the promoter-LUC reporter (pGreenII 0800-*DhPMM-pro*) significantly increased luminescence activity compared to empty vector controls (pGreenII 62-SK). Normalized LUC/REN ratio of activity measurements suggested that NAC, DOF, ERF, and BES1 function as transcriptional activators of *DhPMM* (Figure 6a–d).

## 4. Discussion

Polysaccharides constitute essential bioactive compounds in *D. huoshanense*, with their biosynthesis involving coordinated enzymatic cascades. PMM, an enzyme of great importance in the mannose metabolic pathway, catalyzes the reversible isomerization of mannose-1-phosphate (M1P) to mannose-6-phosphate (M6P), positioning GDP-mannose as the central precursor for polysaccharide formation [34]. Our previous work verified PMM protein participated in polysaccharide biosynthesis pathway after crotonylation, which may enhance PMM’s catalytic activity [33]. Beyond post-translational modifications, transcription factors represent critical regulators of enzymes, thereby critically governing polysaccharide accumulation. These DNA-binding proteins recognize *cis*-elements in target gene promoters to orchestrate spatiotemporal gene expression [35]. As trans-acting regulators, TFs orchestrate multifaceted physiological processes, including growth and development [36], secondary metabolite biosynthesis [37], and responses to biotic/abiotic stresses [38] through precise control of gene expression dynamics. Notably, no transcriptional regulators of *DhPMM* had been characterized prior to this investigation—a significant knowledge gap given PMM’s pivotal metabolic role in *D. huoshanense*.

The yeast one-hybrid screening system is a gold-standard method of interrogating interactions between DNA sequences and DNA-binding proteins, particularly TFs [39]. The efficacy of cDNA library screening critically depends on two pivotal quality metrics: library complexity and average insert size, which collectively determine the coverage of low-abundance transcripts and functional gene representation [40]. Critical cDNA library parameters include a minimum library complexity of ≥1 × 10^6^ CFU and insert size of ≥ 1 kb to ensure comprehensive detection of rare transcripts. Our cDNA library screening achieved a complexity of 3.60 × 10^9^ CFU and an average insert size of 1.5 kb (range of 1–2 kb determined by colony PCR). These optimized parameters collectively satisfied the technical prerequisites for conducting systematic Y1H screening [41] and enabled successful identification of *DhPMM* transcriptional regulators.

High-complexity cDNA library screening enables rapid identification of transcription factors in medicinal plants, as established in prior studies [42,43,44]. In this work, we generated a cDNA library of *D. huoshanense* to capture full-length transcripts of transcription factors. Following BLAST analysis of filtered sequences against the NCBI database, we systematically characterized the domain of candidate genes to identify TFs. It finally revealed four TFs spanning the AAAG-box-combined DOF, G-box-combined NAC, DRE-combined ERF, and BRRE-combined BES1 families, which are key regulatory components warranting prioritization in subsequent studies. Consistent activation of the *DhPMM* promoter by these four TFs identified through Y1H screening combined with dual-LUC assay enlightened a new perspective of cooperative regulation of polysaccharide biosynthesis in *D. huoshanense*, potentially through binding to conserved *cis*-elements.

**Figure 7:**
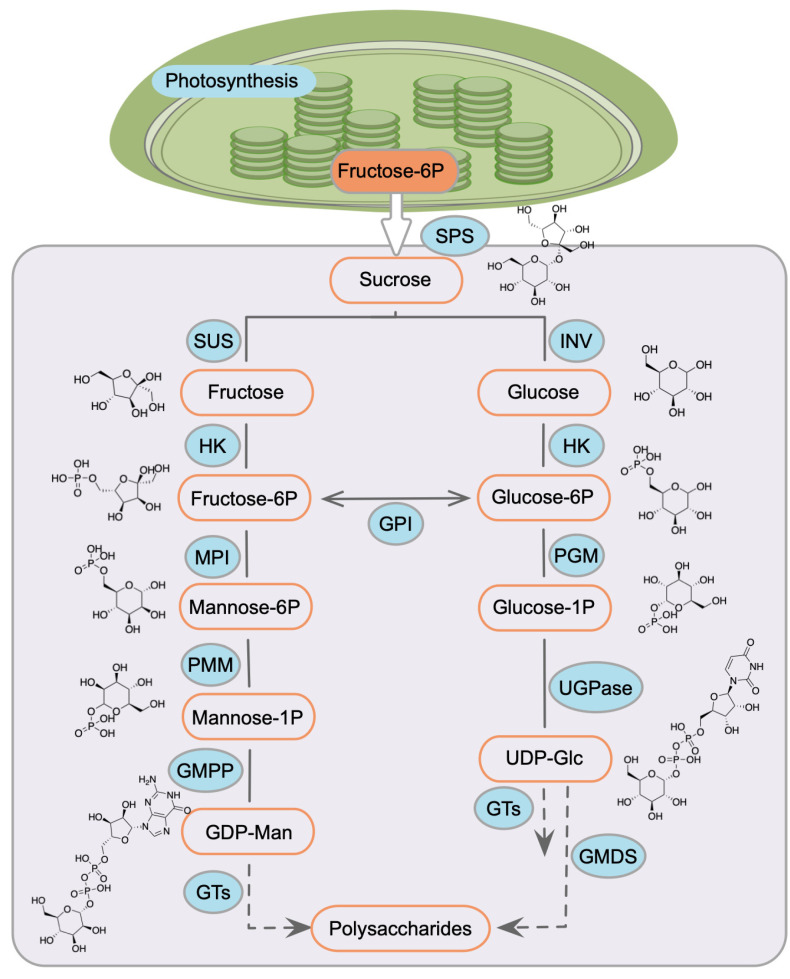
Putative pathway of polysaccharide biosynthesis in *D. huoshanense*. SPS: sucrose phosphate synthase, SUS: sucrose synthase, INV: invertase, HK: hexokinase, GPI: glucose-6-phosphate isomerase, MPI: mannose-6-phosphate isomerase, PGM: phosphoglucomutase, PMM: phosphomannomutase, GMPP: GDP-mannose pyrophosphorylase, GMDS: GDP-Mannose 4,6-Dehydratase, UGPase: UDP-Glc pyrophosphorylase, and GTs: glycosyl transferases.

Based on previous studies in polysaccharide biosynthesis in plants [45,46,47], we proposed polysaccharide biosynthesis in *D. huoshanense* initiates from sucrose conversion via sucrose phosphate synthase (SPS) and then bifurcates into two metabolic branches (Figure 7): (1) the mannose flux pathway: sucrose synthase (SUS) -mediated fructose, PMM-catalyzed M1P; and (2) uridine diphosphate glucose (UDP-Glc) synthesis via SPS or invertase (INV). Glycosyltransferases integrate these monosaccharide units into polymers, with TF-mediated transcriptional regulation serving as a key control point. Our screening identified four TFs regulating *DhPMM* in this cascade. In addition, NAC TF identified in Y1H screening shows homology to *MdNAC5* [48] and *FaNAC035* [49], which bind to promoters of INV, SPS, and SUS to modulate transcriptional activation. Studies also unveiled *ClNAC68* knockout reduced sucrose accumulation by suppressing *ClINV* expression [50], while *FvNAC073* overexpression upregulated *FvSPS1* and downregulated *FvSUS2* to enhance soluble sugar content [51]. Intriguingly, no prior evidence links NAC to PMM transcriptional regulation. Our discovery of a *D. huoshanense*-specific NAC TF represents the first reported regulatory node in mannose metabolism. Plant-specific DOF TFs contain a highly conserved region of 50 amino acid residues with a C2–C2 zinc finger motif [52]. This motif interacts with the *cis*-elements containing a common core (A/T) AAAG or the P-box (TTATGG) in the promoter [53,54]. We found there were abundant (A/T) AAAG motifs in the promoters of *DhPMM* (Table 1). DOFs worked significantly in regulating the expression of genes engaging in saccharides’ metabolism [55], biosynthesis [56], and transport [57]. It has been reported that *ZmDof3* upregulated the expression of SUS to administrate the content of sugar in maize [58]. In tomato, *SlDof22* can play a role by regulating genes in the carbohydrate metabolism pathway, including *PMM* gene [59]. As described above, this DOF TF we screened likely modulates *DhPMM* expression, highlighting its potential as a metabolic engineering target. Additionally, another two TFs (ERF, BES1) showed limited direct polysaccharide links but exhibited metabolic crosstalk. ERF TFs primarily mediated stress responses by binding dehydration-responsive elements (DRE) in target promoters [60]. PtrERF110 activated *PtrSPS4* and further regulated sugar and sterol biosynthesis when exposed to cold stress [61]. BES1, governed development via brassinosteroid signaling [62] and stress-induced developmental plasticity [63]. Collectively, the *DhPMM* promoter contains multiple binding motifs for all four TFs (Figure 1b). This strongly suggests these four TFs combinatorial regulatory potential in modulating *DhPMM* expression—a prerequisite for enhancing polysaccharide accumulation in *D. huoshanense*.

## 5. Conclusions

This study systematically identified upstream genes including TFs with potential to regulate the expression of *DhPMM.* The 2.1 kb *DhPMM* promoter, enriched with various *cis*-elements, was cloned to construct high-complexity cDNA library (3.60 × 10^9^ CFU). Y1H and dual-LUC assays validated interactions between four TFs (DOF, NAC, ERF, BES1 families) and *DhPMM*, which may be linked to regulating polysaccharide biosynthesis. This first-reported *D. huoshanense* cDNA library provides a foundational resource for bridging transcriptional regulation with active constituent’s content, offering insights for the sustainable utilization of non-model medicinal plant.

## Figures and Tables

**Table 1:** *Cis*-elements in sequence of *DhPMM* promoter.

*Cis*-Element	Number	Sequence	Function
AAAG-box	11	AAAG	common cis-acting element in promoter and enhancer regions
ABRE	3	ACGTG	ABA-responsive element
as-1	1	TGACG	cis-acting element involved in environment responsiveness
AuxRE	1	TGTCTCAATAAG	part of an auxin-responsive element
BRRE	3	CGTGT/CG	cis-acting element involved in abiotic stress
CAAT-box	38	CA/CAAT	common cis-acting element in promoter and enhancer regions
DRE	1	ACCGAGA	cis-acting element involved in abiotic stresses responsiveness
ERE	2	ATTTCATA	ethylene response element
F-box	1	CTATTCTCATT	involved in regulation of gene transcription
G-box	4	CACGTC/G/T	cis-acting regulatory element involved in light responsiveness
GT1	2	GGTTAA	light responsive element
I-box	1	GATAAGGTG	part of a light responsive element
MYB	1	TAACTG	MYB element
MYC	5	CAAT/TGTG	MYC element
TATA-box	20	TATA	core promoter element around −30 of transcription start site
TATC-box	2	TATCCCA	cis-acting element involved in gibberellin-responsiveness
TCA-element	2	CCATCTTTTT	cis-acting element involved in salicylic acid responsiveness
W box	1	TTGACC	WRKY motif

**Table 2:** Annotation of positive sequences from the Y1HGold [pAbAi-*DhPMM*].

Number	Gene Bank	Annotation Information	Species	TF Family
D1	XP_020700119.1	30S ribosomal protein S20, chloroplastic isoform X1	*Dendrobium catenatum*	NAC
D2	XP_020682760.1	dof zinc finger protein 3-like isoform X1	*Dendrobium catenatum*	DOF
D3	XP_020701931.1	reticulon-like protein B12	*Dendrobium catenatum*	
D4	XP_020674477.1	UPF0565 protein C2orf69 homolog isoform X1	*Dendrobium catenatum*	ERF
D5	XP_020699932.1	protein BRICK 1	*Dendrobium catenatum*	
D6	XP_020701822.1	macrophage migration inhibitory factor homolog isoform X2	*Dendrobium catenatum*	BES1
D7	QGJ03755.1	transport inhibitor response 1-like protein	*Vanilla planifolia*	
D8	XP_020671916.1	peroxiredoxin-2C	*Dendrobium catenatum*	
D9	PKU62163.1	ATP synthase subunit delta, mitochondrial	*Dendrobium catenatum*	
D10	XP_020690022.2	uncharacterized protein LOC110105016 isoform X1	*Dendrobium catenatum*	
D11				

## Data Availability

The datasets generated during the current study are openly available in Sequence Read Archive (SRA) of NCBI (https://www.ncbi.nlm.nih.gov, accessed on 20 August 2025) under the access numbers SRR32943785, SRR32945542, SRR32946154, SRR32944933, SRR32946815, SRR32946151, SRR32946884, SRR32946904, SRR32946921, SRR32946924, and SRR32947996. The associated BioProject and BioSample numbers are PRJNA1245079 and SAMN47738843 (https://www.ncbi.nlm.nih.gov/sra/PRJNA1245079, accessed on 20 August 2025).

## Supplementary Materials

The following supporting information can be downloaded at: https://www.mdpi.com/article/10.3390/biom15091251/s1. Figure S1: Electrophoretic diagram of mRNA (a) and cDNA library (b); Figure S2: Quality identification of the constructed primary and secondary cDNA libraries title.

## Abbreviations

The following abbreviations are used in this manuscript:

AbA	Aureobasidin
ABRE	ABA-responsive element
AMP	Ampicillin
AP2/ERF	APETALA2/Ethylene Response Factor
BES1	Bri1-ems-suppressor 1
BRRE	Brassinosteroid-responsive element
CFU	Colony-forming units
cDNA	Complementary DNA
DRE	Dehydration-responsive element,
DOF	DNA binding with one zinc finger
ERF	Ethylene response factor
GDP-Man	Guanosine diphosphate mannose
GTs	Glycosyltransferases
HK	Hexokinase
INV	Invertase
LB	Luria–Bertani medium
M1P	Mannose-1-phosphate
M6P	Mannose-6-phosphate
MPI	Mannose-6-phosphate isomerase
MYC	Myc transcription factor family
NAC	Nam, ataf, and cuc
PCR	Polymerase chain reaction
PEG	Polyethylene glycol
PGM	Phosphoglucomutase
PMM	Phosphomannomutase
SD	Synthetic-defined medium
SMART	Switching mechanism at 5′ end of RNA template
SPS	Sucrose phosphate synthase
SUS	Sucrose synthase
TF	Transcription factor
UDP-Glc	Uridine diphosphate glucose
Y1H	Yeast one-hybrid
YPDA	Yeast extract peptone dextrose adenine medium
