# Supplementary material for: Construction of Yeast One-Hybrid Library of Dendrobium huoshanense and Screening of Potential Transcription Factors Regulating DhPMM Gene Expression
Source: Biomolecules. 2025 Aug 29;15(9):1251. doi: 10.3390/biom15091251 (PMC12467618; doi:10.3390/biom15091251)
Supplement: Supplementary file 1 [file biomolecules-15-01251-s001.zip › biomolecules-3806831-supplementary.pdf]

Supplementary Materials

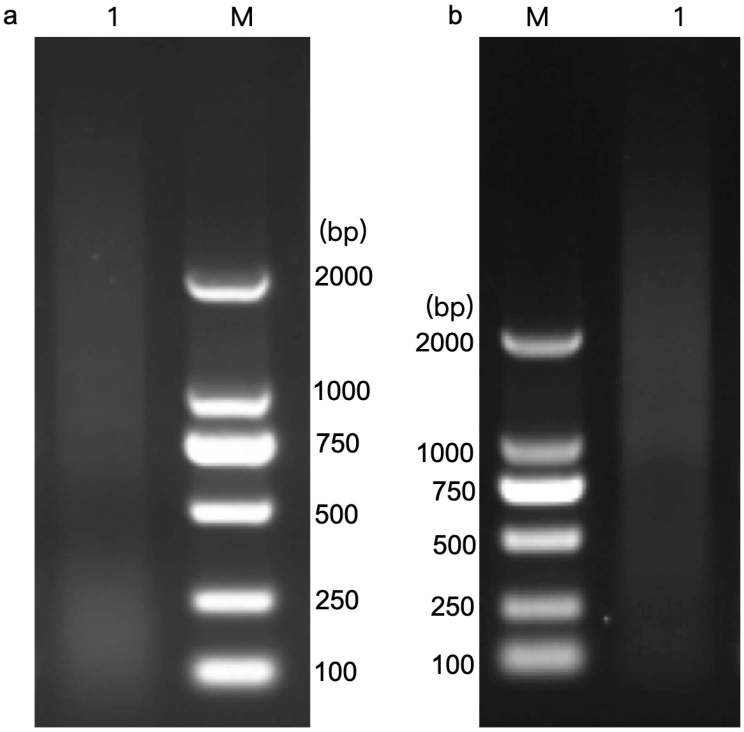

Figure S1. Electrophoretic diagram of mRNA (a. Lane 1: mRNA) and cDNA (b. Lane 1: cDNA).

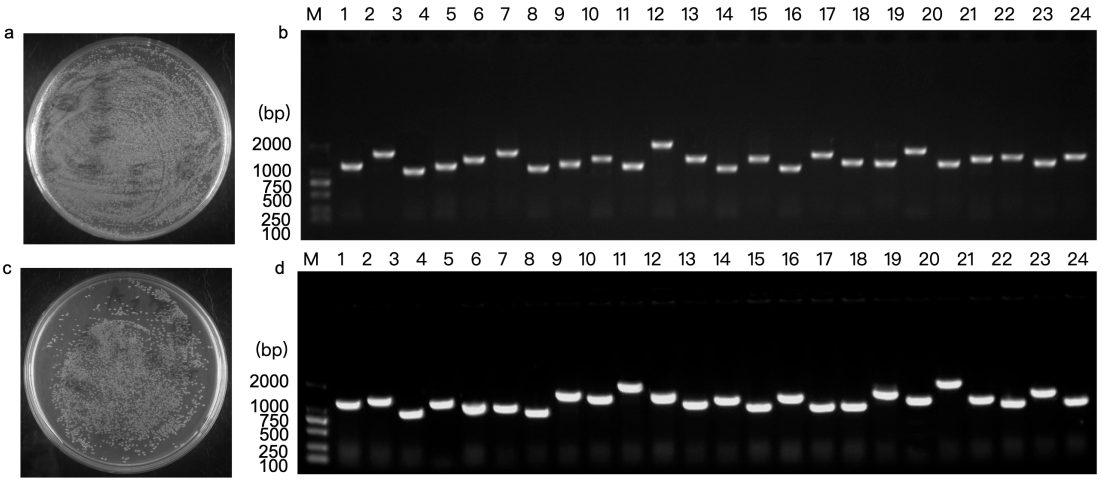

Figure S2. Quality identification of the constructed primary and secondary cDNA libraries. (a). Capacity of the primary cDNA library (1.44×10^7 CFU). (b). The size of 24 clones in the primary cDNA library. Lanes 1-24: 24 randomly selected clones. (c). Capacity of the secondary cDNA library (1.20×10^7 CFU). (b). The size of 24 clones in the secondary cDNA library. CFU: PCR colony-forming units. Lanes 1-24: 24 randomly selected clones.
